# Development of Non-Viral, Trophoblast-Specific Gene Delivery for Placental Therapy

**DOI:** 10.1371/journal.pone.0140879

**Published:** 2015-10-16

**Authors:** Noura Abd Ellah, Leeanne Taylor, Weston Troja, Kathryn Owens, Neil Ayres, Giovanni Pauletti, Helen Jones

**Affiliations:** 1 James L. Winkle College of Pharmacy, University of Cincinnati, Cincinnati, OH, 45267, United States of America; 2 Faculty of Pharmacy, Assiut University, 71515, Assiut, Arab Republic of Egypt; 3 Department of Chemistry, University of Cincinnati, Cincinnati, OH, 45221, United States of America; 4 Divisions of General and Thoracic Surgery and Reproductive Sciences, Cincinnati Children’s Hospital Medical Center, Cincinnati, OH, 45229, United States of America; Chinese Academy of Sciences, CHINA

## Abstract

Low birth weight is associated with both short term problems and the fetal programming of adult onset diseases, including an increased risk of obesity, diabetes and cardiovascular disease. Placental insufficiency leading to intrauterine growth restriction (IUGR) contributes to the prevalence of diseases with developmental origins. Currently there are no therapies for IUGR or placental insufficiency. To address this and move towards development of an in utero therapy, we employ a nanostructure delivery system complexed with the IGF-1 gene to treat the placenta. IGF-1 is a growth factor critical to achieving appropriate placental and fetal growth. Delivery of genes to a model of human trophoblast and mouse placenta was achieved using a diblock copolymer (pHPMA-b-pDMAEMA) complexed to hIGF-1 plasmid DNA under the control of trophoblast-specific promoters (Cyp19a or PLAC1). Transfection efficiency of pEGFP-C1-containing nanocarriers in BeWo cells and non-trophoblast cells was visually assessed via fluorescence microscopy. *In vivo* transfection and functionality was assessed by direct placental-injection into a mouse model of IUGR. Complexes formed using pHPMA-b-pDMAEMA and CYP19a-923 or PLAC1-modified plasmids induce trophoblast-selective transgene expression *in vitro*, and placental injection of PLAC1-hIGF-1 produces measurable RNA expression and alleviates IUGR in our mouse model, consequently representing innovative building blocks towards human placental gene therapies.

## Introduction

Inappropriate placental development affecting up to 6% of all pregnancies is associated with multiple pathologies and accounts for a 75% of all cases of intra-uterine growth restriction [1]. Intra-uterine growth restriction (IUGR) is recognized as the failure of an infant to achieve their full growth potential in utero and is the second leading cause of perinatal morbidity and mortality [2]. Furthermore, the association between IUGR and the development of adult diseases such as obesity, type 2 diabetes and cardiovascular disease in later life is well established [3]. Insulin-like Growth Factors (IGF1 &2) have an important role in fetoplacental growth throughout gestation [4]. The concept of growth factor deficiency in placental insufficiency is supported by experimental evidence from specific growth factor null transgenic mice. These knock out mice models provided proof of concept that the IGF system is a major regulatory system for growth and development of the fetus [5, 6]. There is clinical evidence that placental insufficiency is associated with deficiencies in growth factors, abnormalities in maternal- fetal placental vascular system (angiogenesis and vasculogenesis) as well as placental nutritional transport systems.

There is currently no treatment for placental insufficiency or IUGR except premature delivery, which can result in developmental delays and higher incidence of infant mortality. Previous experiments using adenovirus-mediated gene transfer to the placenta modulates placental function and fetal growth in vivo [7, 8]. These studies have also shown that transgene expression is minimal but still occurs in the fetus or mother following placental delivery of gene therapy [9]. However, the development of non-viral tissue-specific transgene delivery systems ensures the safe application of gene therapy in the developing human placenta. Despite evidence that nanoparticles and environmental nano-sized materials cross the placental barrier [10], there is no current literature investigating the use of nanoparticles for gene delivery to the placenta. Historically, nanoparticles have been designed for imaging, targeting, and destruction of unwanted cells such as tumors [11]. These nanoparticles are unsuitable for placental use due to undesired accumulation within the tissue and toxicity of the particles. However, the development of polymer-based biodegradable nanoparticles [12] permits non-viral gene delivery leading to enhanced cell and organ function. In these studies, we employ poly[2-hydroxypropyl) methacrylamide (HPMA), a water soluble polymer that increases delivery of IGF-1 through absorptive endocytosis and is biocompatibile [13] non-immunogenic [14] and specific. Purified plasmid DNA containing a sequence for IGF-1 is complexed by poly(2-(N,N-dimethylamino)ethyl methacrylate (DMAEMA), a tertiary amine that acts as a weak base capable of being protonated at biological pH [15]. Together these co-polymers provide a non-viral nanoparticle alternative for gene transfer into the placenta. We have previously characterized complex formation using these co-polymers [16].

The ideal vector for gene transfer should result in high tissue-specific transgene expression with minimal off-target gene delivery. Tissue or cell-specific promoters reduce off-target gene expression and studies have identified both CyP19a and PLAC1 as genes with trophoblast-specific promoters [17, 18]. Here we demonstrate efficient, targeted gene delivery to a human trophoblast cell line using nanoparticle-mediated IGF-1 delivery. In a mouse model of surgically-induced placental insufficiency, we are able to detect expression of human IGF-1 in placenta and maintenance of fetal growth.

## Materials and Methods

### Copolymer synthesis and Polymerization

**Fig 1:**
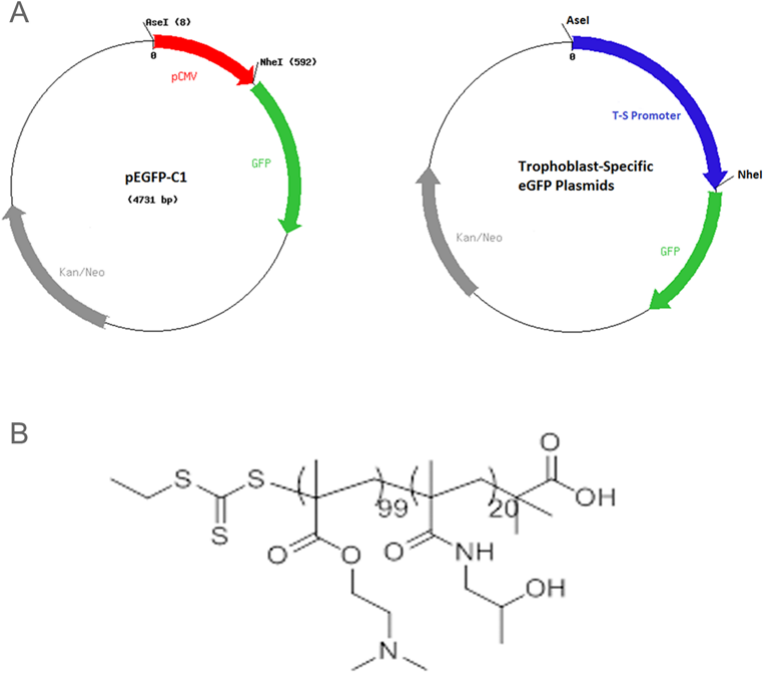
(A) The HPMA-DMAEMA copolymer used for DNA delivery in both *in vitro* and *in vivo* studies. (B) Maps of the CMV-eGFP and Trophoblast-specific plasmids.

All starting materials were purchased from Aldrich at the highest purity available and used as received unless specified otherwise. Synthesis of *S-1-ethyl-S’-(α’*,*α’-dimethyl-α”-acetic acid)trithiocarbonate (EDMAT)* was adapted from the procedure described by Convertine et al. [19]. Synthesis of 2-Hydroxypropyl methacrylamide (HPMA) monomer was adapted from the procedure described by Rowe et al. [20]. Polymerization of HPMA with RAFT agent was adapted from the procedure described by Rowe et al. [20], briefly, the EDMAT RAFT agent (0.0314 g, 0.001 mol), HPMA (4.0 g, 0.03 mol), AIBN (0.0023 g, 0.0001 mol), and tert-butanol (27 mL, 0.28 mol) were added to a 100 mL round-bottom flask equipped with a stir bar. The reaction was placed in an ice bath and the reaction mixture was purged under nitrogen for 45 min and left under an N2 atmosphere. The reaction was transferred to an 80°C oil bath and allowed to react for 6 h, sampling every hour. The reaction was quenched by exposure to air and precipitated from ether to yield a pale yellow polymer. Isolated yield: 40%. PDI = 1.24. Mn = 13000 g/mol. 1H NMR (CDCl3): 1.1 (d, 76H), 1.3 (s, 3H), 3.19 (t, 1H), 3.95 (d, 2H), 6.58 (br, 1H). FT-IR (cm-1): ν(OH) = 3323.3 broad, ν(CH = CH2) = 2979.9 and 2291.1, ν (NHC = O) = 1672.4. Polymerization of DMAEMA with HPMA macro-RAFT agent was adapted from the procedure described by Duvall et al. [21]. The HPMA macro-RAFT agent (0.07 mmol), 2-(dimethylamino)ethyl methacrylate (DMAEMA) (0.012 mol), 2,2’-azobis(2-methylpropionitrile) (AIBN) (0.077 mmol), and dimethylformamide (0.15 mol) were added to a 100mL round-bottom flask equipped with a stir bar. The reaction was placed in an ice bath and purged under nitrogen for 45 min and left under an N2 atmosphere. The reaction was transferred to a 65°C oil bath and allowed to polymerize for 6 h. The reaction was quenched by exposure to air and precipitated from ethyl ether:pentane (50:50). The resulting copolymer structure is shown in Fig 1.

### Plasmid Design

Two fragments of the human CyP19a promoter, corresponding to the -923 and -501 fragments described by Kamat et al. [22] were cloned into the pEGFP-C1 plasmid replacing the CMV promoter (Fig 1A, Promega, Madison, WI). PLAC1 promoter (GeneCopia, Rockville, MD) was also cloned into the pEGFP-C1 plasmid (Fig 1B). For functional studies, the human IGF-1 (hIGF-1) sequence was cloned into the constructs instead of the GFP. Following sequence confirmation and expansion, plasmids were purified using an Endo-Free Plasmid Maxi Kit (Qiagen), per manufacturer’s protocol. The yield, purity, and integrity of the prepared plasmids were evaluated with a nanodrop (Thermo Scientific, Waltham, MA) and by gel electrophoresis.

### Cell Culture

The BeWo Choriocarcinoma cell line was obtained from ATCC and cultured in Ham’s F12 nutrient mix supplemented with 10% fetal calf serum and 2% penicillin/streptomycin (Invitrogen,). Human Uterine Fibroblasts (a kind gift from Dr. Sanjoy Das, Cincinnati Children’s Hospital Medical Center) and Human Embryonic Kidney 293 cells were maintained in high glucose DMEM (Invitrogen) supplemented with 20% fetal calf serum, 2% penicillin/streptomycin and 1% sodium pyruvate. Human Placental Microvascular Endothelial cells were maintained in EGM-2 (Lonza Inc. Williamsport, PA). All cells were routinely maintained at 37°C under 5% CO2.

### Transfection

eGFP- or hIGF-1-containing nanoparticles fabricated in PBS at an N/P ratio of 4 [16] were used for transfection experiments. To confirm specificity, cells were grown on glass coverslips and incubated with the uncomplexed DNA plasmid or nanoparticle complexes overnight in serum-free medium, following addition of serum, cells were cultured for a further 72 hours. Control cells were incubated with either nanoparticles including the CMV-eGFP to compare expression levels with a constitutive promoter or uncomplexed plasmid eGFP to demonstrate the benefits of the nanoparticle for DNA delivery. After incubation with DNA or Nanoparticle for 96 hours cells were fixed in ice cold methanol, washed and mounted with Prolong Gold antifade reagent with Dapi. Expression was assessed by fluorescent microscopy (Nikon Eclipse 80i). Cells exposed to hIGF-1 nanoparticles were harvested for RNA extraction to assess gene expression.

### Cell viability

BeWo proliferation was assessed as previously described [23] in triplicate using the crystal violet assay across 4 different passages following incubation with either the GFP-containing nanoparticle or naked GFP plasmid for 96 hours. Apoptosis was investigated using immunocytochemistry with cells plated onto chamber slides and anti-Caspase 3 antibodies. Caspase-3 positive cells were counted and expressed as a ratio of total cell number per high powered field using a Nikon microscope and Elements software (Nikon).

### Immunocytochemistry

Anti-Caspase-3 antibody was applied to cells in 0.2% FSG/PBS and allowed to bind overnight at 4°C in a humidified atmosphere. Following rinses, FITC conjugated secondary antibody (Vector Laboratories Inc, Burlinghame, CA, USA) was applied to the cells for 1 h at room temperature in a humidified atmosphere. The cells were rinsed in PBS and deionised water and mounted in Vectashield mounting medium with DAPI (Vector Laboratories).

### *In vivo* delivery

All animal procedures were performed under protocols OCO3024 and 3DO2011 approved by the Institutional Animal Care and Use Committee of Cincinnati Children’s Hospital Medical Center. Timed pregnant C57 Bl/6J mice (aged 7–9 weeks) were divided into 4 groups: 1) Control: Sham operated; 2) uterine artery branch ligation (UABL); 3) UABL with intra-placental injection of NP-PLAC1-HuIGF-1 (63ng DNA); and 4) UABL with intra-placental injection of NP-CyP19a-923-HuIGF-1 (14ng DNA). At gestational day 16, through laparotomy, UABL was performed and treatment was administered as previously described to no more than 4 fetuses per litter [7, 8, 24]. Placentas from unligated and untreated pups within litters were used as internal controls (IC). Cesarean-section was performed on day 20; pups and placentas were weighed and snap frozen or fixed in PFA for further analysis. Each n number represents one Dam. Sham birth weights represent the mean of the whole litter, IC birth weights are the mean weights of littermates not undergoing ligation or treatment.

### Reverse Transcription quantitative Polymerase Chain Reaction (RT-qPCR)

Total RNA was isolated from cultured cells or placentas using the RNeasy kit (Qiagen, Valencia, CA) per manufacturer’s protocol. cDNA was synthesized from 1 μg of total RNA using the High Capacity cDNA Reverse Transcription kit (Applied Biosystems, Foster City, CA) following suppliers instructions. SYBR green assays were designed to span intron/exon boundaries when possible. Oligonucleotide primers were aligned against the human genome by Primer-BLAST (www.NCBI.org) to ensure specificity. Gene expression was assayed in duplicate as previously described [7], Oligonucleotide primer sequences are as listed in Table 1.

**Table 1:** Oligonucleotide primers for RTqPCR.

Oligonucleotides	Sequences (5′‐3′)	Amplicon (bp)	Intron (bp)
Hu TBP F	GAACCACGGCACTGATTTTC		
Hu TBP R	TGCCAGTCTGGACTGTTCTTC	**77**	**2285**
Hu ACTB F	CGC GAG AAG ATG ACC CAG		
Hu ACTB R	TAG CAC AGC CTG GAT AGC AA	**75**	**441**
Hu IGF1 F	TTT TGT GAT TTC TTG AAG GTG A		
Hu IGF1 R	CGG TCC AGC CGT GGC AGA	**104**	**4519**
Mo ACTB F	CTA AGG CCA ACC GTG AAA AGA T		
Mo ACTB R	CAC AGC CTG GAT GGC TAC GT	83	454

### Placental Histology

Isolated placentas were fixed in 10% buffered formalin overnight at 4°C, processed placental tissues were embedded in paraffin, and 5μm sections cut. Serial sections were deparaffinized, rehydrated and stained with Hematoxylin and Eosin following standard protocols. Following dehydration steps, sections were mounted and placental morphology observed by light microscopy (Nikon, Melville, NY) and assessed for inflammation or infiltration by analyzing 10 High Powered Fields in 5 sections from 3 placentas in each group.

### Data presentation and statistics

Data are presented as means ± SEM. Statistical analysis was performed using ANOVA with post hoc Tukey’s test *p<0.05, **p<0.01,

## Results

### Reporter Gene Expression

**Fig 2:**
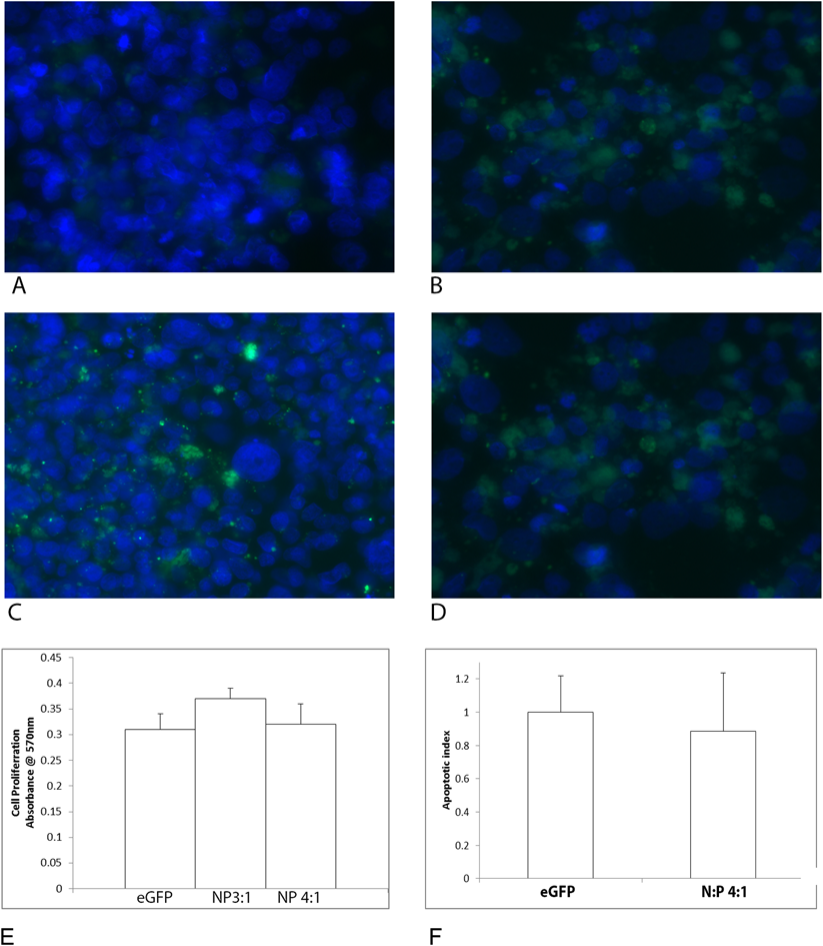
Representative images of (A) GFP expression following transfection of BeWo with plasmid alone or (B) CMV-eGFP nanoparticles demonstrate greater transgene expression following complex formation than plasmid alone. (C) GFP expression in BeWo cells after transfection with nanoparticles containing PLAC1-GFP or (D) CyP19a-923-GFP for 4 days, resulted in comparable transgene expression to the global CMV promoter, similar results were seen on 4 different passages. Cell nuclei are stained with Dapi (blue). Proliferation levels (E) and apoptosis levels (F) in BeWo cells were not changed when cells were incubated with nanocomplexes for 48 hours compared to BeWo cells incubated with eGFP plasmid alone for the same time period, mean +/- SEM, n>4 passages per treatment.

GFP expression was higher in the BeWo cells incubated with any of the nanoparticles containing trophoblast-specific promoters than those incubated with the uncomplexed DNA. In the BeWo cells, GFP expression levels under the control of the trophoblast-specific promoters, CyP19a-923 and PLAC1, were comparable with the expression seen after transfection with the GFP under the control of the CMV promoter (Fig 2).

### Cell Viability

To assess the effects, if any, of the nanoparticle on cell viability we analyzed proliferation and apoptosis in the BeWo cell line. Proliferation rates and apoptotic indices remained at control levels when BeWo cells were incubated with either DNA plasmids alone or complexed DNA nanoparticles for 96 hours (Fig 2E & 2F).

### Cell Specificity

**Fig 3:**
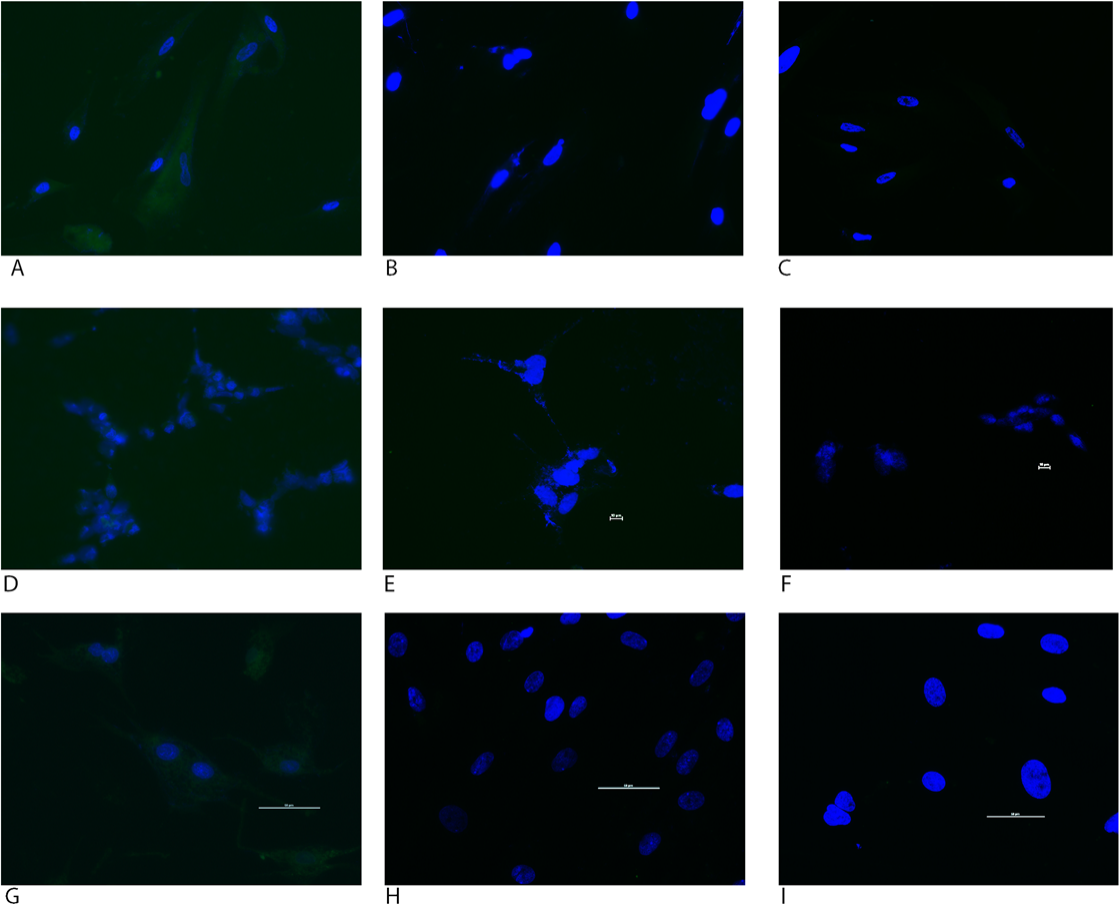
GFP expression in Human uterine fibroblasts after transfection with nanoparticles containing (A) CMV-GFP, (B) PLAC1-GFP or (C) CyP19a-923-GFP. HEK293 cells transfected with (D) CMV-GFP, (E) PLAC1-GFP or (F) CyP19a-923-GFP demonstrated low but visible transgene expression under the CMV promoter but no GFP expression with the trophoblast-specific promoters. GFP expression in HPMVECs cells after transfection with nanoparticles containing (G) CMV-GFP, (H) PLAC1-GFP or (I) CyP19a-923-GFP. Representative images seen on 4 different passages for each cell type. Cell nuclei are stained with Dapi (blue).

Expression of GFP was seen in human uterine fibroblasts following incubation with nanoparticles containing GFP under the control of the CMV promoter (Fig 3A), but no GFP expression was observed in fibroblasts when the trophoblast-specific promoters were used (Figs 3B & 2C). Similarly, Human Placental Microvascular Endothelial cells expressed GFP following transfection with the nanoparticle containing the CMV-GFP plasmid as seen in Fig 3G but no GFP was expressed following transfection with either PLAC1-GFP or the CyP-19-923-GFP (Figs 3H & 2I). Interestingly, HEK293 cells showed minimal GFP expression following transfection with the CMV-GFP nanoparticles (Fig 3D) but again showed no expression when the trophoblast-specific promoters were used (Figs 3E & 2F).

### Human IGF-1 expression

IGF-1 expression was measured by real-time qPCR following treatment of BeWo cells for 96 hours. Significant (p = 0.003, N≥ 4 per group) increases in IGF-1 expression (RQ) were found following treatment with both NP-Cyp19a-hIGF-1 (181±67) and NP-PLAC1-hIGF-1 (225±72) polyplexes compared to cells treated with plasmid alone (1.38±0.65).

### Birthweight

**Fig 4:**
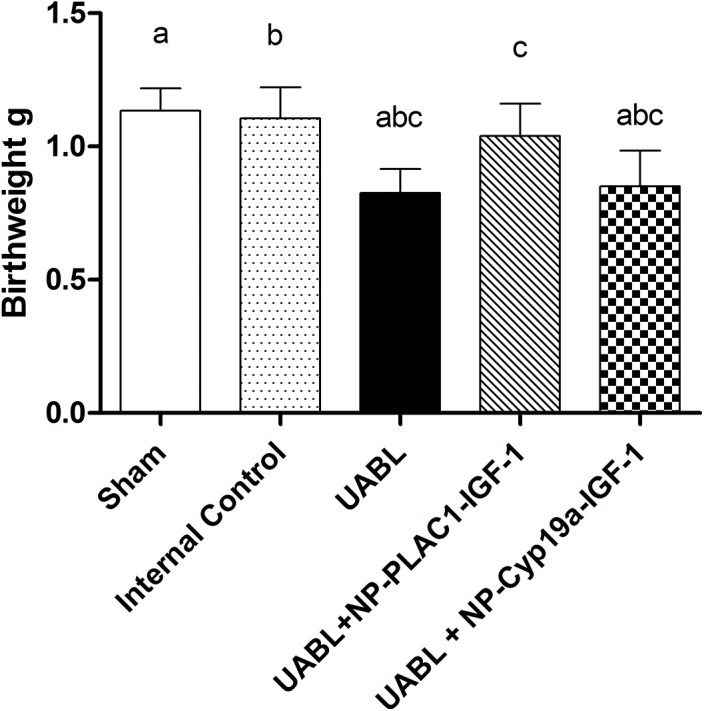
Offspring birthweights at delivery were the same in Sham-operated, Internal control and UABL + PLAC1-HuIGF-1 nanoparticle treated group, however the Uterine Artery Branch Ligation, and Cyp19a-HuIGF-1 nanoparticle treated groups had significantly lower birthweights. N>6 dams per group, ANOVA<0.001, post-hoc Tukeys *<0.05, **<0.01.

Offspring from all treatments were delivered and viable at day 20 of gestation by caesarian section. Numbers of pups per litter at delivery were not altered by UABL or nanoparticle treatment compared to litter size recorded at time of surgery. Offspring weights of those whose placentas had received the PLAC1-IGF-1(1.04±0.05) nanoparticle showed significantly (p<0.05, ANOVA, n> 6 per group) higher birth weights than those in the UABL group (0.82±0.04) or those who underwent treatment with the CyP19a-923-IGF-1 (0.85±0.65) nanoparticle (Fig 4A). PLAC1-IGF-1 nanoparticle-treated pups weighed the same as the sham-operated and internal controls (1.13±0.04, and 1.11±0.05, Fig 4).

### Transgene expression *in vivo*

Human IGF-1 gene expression was detectable by qPCR in the placentas injected with either PLAC1 or CyP19a driven IGF-1 DNA-polymer complexes (RQ values of 243± 60 and 213± 45, respectively, n≥5 for each treatment). A placental sample from previous studies with Ad-hIGF-1 injection [4] was used as the standard (RQ = 1) as no expression of Human IGF-1 was found in, sham, UABL nor internal control placentas taken from the opposite horn of the uterus from the injection site or fetal liver samples from fetuses whose placentas were NP-PLAC1-hIGF-1 or NP-CYP19-hIGF-1 treated.

### Placental Characteristics

**Fig 5:**
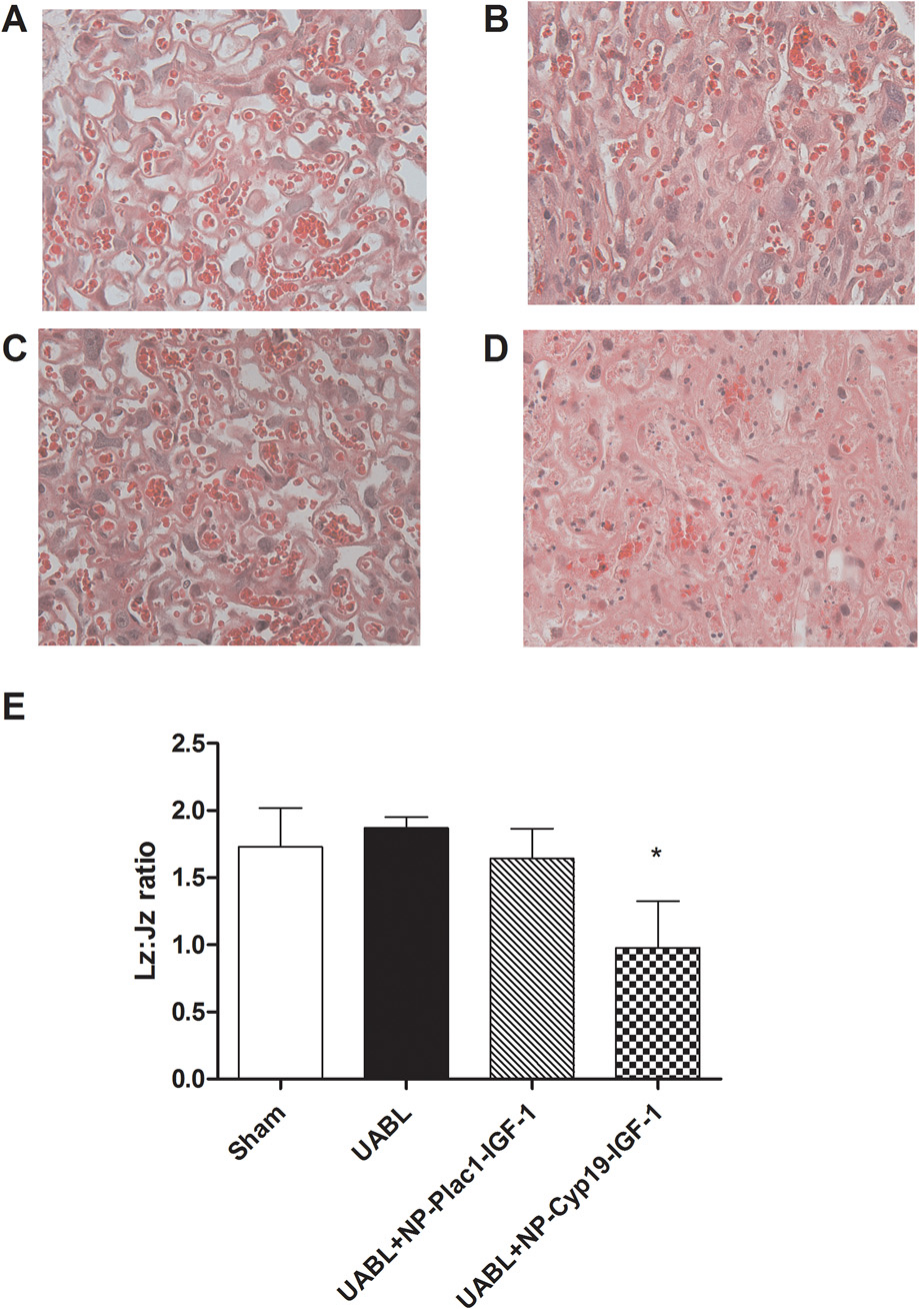
Placental morphology in the Labyrinthine (L) zone in (A) Sham, (B) UABL,(C) NP-PLAC1-hIGF-1treated groups is similar whereas differences in morphology can be seen in the NP-Cyp19a-hIGF-1 (D) treated group. (Magnification 40X). (E) The ratio of placental Junctional zone (Jz) area to Labyrinthine zone (Lz) area demonstrates significant expansion of the junctional zone in placentas treated with NP-Cyp19a-hIGF-1.

There were no differences in placental weights between sham and UABL groups as previously reported by our group [24] or between sham and any of the nanoparticle-treated groups (0.09g ± 0.01 vs. 0.08g ± 0.02 vs. 0.10g ± 0.03, n>5 per group). Labyrinth depth was significantly reduced in the UABL group as previously reported, however in the NP-PLAC1-IGF-1 (1.15mm ± 0.85) treated placentas it was the same as the sham group (1.07mm ± 0.09) and significantly increased compared to the UABL group (0.68mm ± 0.65) (p<0.05, n = 5 per group). There was no evidence of inflammation or immune cell infiltration seen in the sham (Fig 5A), UABL (Fig 5B) or NP-PLAC1-IGF-1 (Fig 5C) injected placentas following H&E staining. However, NP-CyP19a-hIGF-1 placentas showed evidence of morphological disturbance with disorganization in the labyrinth zone (Fig 5D) and expansion of the junctional zone as demonstrated by the altered ratio of labyrinth to junctional zone (Fig 5E), as shown previously in some transgenic strains [25].

## Discussion

This is the first study to successfully deliver a human transgene into a mouse placenta in vivo and human trophoblast in vitro using non-viral mechanisms. Importantly, we demonstrate that delivery of IGF-1 via the nanoparticle was able to express sufficient transgene to restore normal fetal growth in a mouse model of placental insufficiency and fetal growth restriction. Currently no therapy exists for treating placental insufficiency in cases of fetal growth restriction and the development of an in utero therapy could alter the fetal growth trajectory resulting in appropriate birthweight and reduction of clinical outcomes associated with placental insufficiency.

In vitro data in various human cell types demonstrate that incorporation of the plasmid DNA into any of the nanocomplexes, either with the CMV promoter in all cell types or the trophoblast-specific promoters in the BeWo cells, significantly increases GFP expression. Furthermore, in the BeWo cells, IGF-1 expression levels are significantly increased when the plasmids are delivered incorporated within the polyplex compared to just plasmid alone. These increases indicate that polyplex formation leads to preferential uptake which is likely due to a reduction in negative charge compared to plasmid DNA alone and the interaction of the polyplex with negatively charged cell membranes.

For proof-of-concept trials in a model of human trophoblast, GFP expression in the BeWo cell line was comparable in cells exposed to either DNA-nanocomplexes under control of the CMV promoter or trophoblast-specific promoter complexes. This observation is significant because reduced expression under the specific-promoters could impact the levels of complexes needed for treatment.

The placenta is composed of multiple cell types including trophoblast, mesenchymal cells and microvascular endothelial cells. Toward term, placental microvascular endothelial cells are in very close proximity to trophoblast cells [26]. This proximity highlights the importance of cell–specific gene expression to avoid off-target cell delivery of nanoparticles and gene expression in the placenta. Expression of GFP under the control of the CMV promoter, but not the trophoblast-specific promoters, confirms cell specificity even in cells native to the placental environment such as Human Placental Microvascular Endothelial cells and Human Uterine Fibroblasts. To investigate cell specificity in a non-placental cell type, Human Embryonic Kidney 293 cells were transfected with vectors containing trophoblast-specific promoters and showed no GFP expression.

Cell proliferation and apoptosis rates in BeWo cells were unaffected by the incubation with the nanoparticles delivering GFP indicating no evidence of effects on cell viability from the polymer complex or the transfection protocol. Further experiments will need to be performed on primary human trophoblasts and syncytiotrophoblast in the future.

In vivo, we have previously demonstrated that intra-placental injection of adenoviral-mediated IGF-1 therapy can influence placental growth and function [7, 10]. In the current study we have shown that by using the DNA-polyplexes under the control of the PLAC1 promoter we achieve human IGF-1 expression in a mouse placenta with no expression in fetal liver. Furthermore, this therapy reestablishes normal fetal growth in growth restricted fetal mice. RNA levels of human IGF-1 following PLAC1-IGF-1 nanoparticle delivery were significantly higher than those following adenoviral-mediated delivery under control of the global CMV promoter. One shortfall of our study was the we did not assess the impact of direct placental injection of IGF-1 without nanoparticle encapsulation and will investigate this in future studies to highlight the benefit of nanoparticle use. Following collection, processing and histological assessment of a subset of placentas, we did not see any abnormal morphology in the placentas injected with PLAC1-IGF-1 nanoparticle or evidence of inflammatory reaction suggesting the therapy is not detrimental to normal placental morphological development in late gestation. Interestingly, we see evidence of placental morphological dysregulation [27] in the Cyp19a-hIGF-1 injected placentas. Further investigations into this observation are necessary, including assessing protein levels of IGF-1 produced by the therapy.

Future studies will be necessary to assess long-term gene expression following nanoparticle delivery. DNA complexes fabricated in PBS using pHPMA-*b*-pDMAEMA and PLAC1-modified expression plasmids induce trophoblast-selective transgene expression in vitro in a human trophoblast model and in vivo resulting in IGF-1 expression capable of maintaining normal fetal growth. Consequently, this approach represents innovative building blocks towards human placental gene therapies in vivo.
